# Differential response of kabuli and desi chickpea genotypes toward inoculation with PGPR in different soils

**DOI:** 10.3389/fmicb.2015.00859

**Published:** 2015-08-25

**Authors:** Asma Imran, Muhammad S. Mirza, Tariq M. Shah, Kauser A. Malik, Fauzia Y. Hafeez

**Affiliations:** ^1^National Institute for Biotechnology and Genetic EngineeringFaisalabad, Pakistan; ^2^Plant Breeding and Genetic Division, Nuclear Institute for Agriculture and BiologyFaisalabad, Pakistan; ^3^Department of Biological Sciences, Forman Christian College UniversityLahore, Pakistan; ^4^Department of Biological Sciences, COMSATS Institute of Information TechnologyIslamabad, Pakistan

**Keywords:** chickpea, *Ochrobactrum ciceri*, *Mesorhizobium*, PGPR, nodulation

## Abstract

Pakistan is among top three chickpea producing countries but the crop is usually grown on marginal lands without irrigation and fertilizer application which significantly hampers its yield. Soil fertility and inoculation with beneficial rhizobacteria play a key role in nodulation and yield of legumes. Four kabuli and six desi chickpea genotypes were, therefore, evaluated for inoculation response with IAA-producing *Ochrobactrum ciceri* Ca-34^T^ and nitrogen fixing *Mesorhizobium ciceri* TAL-1148 in single and co-inoculation in two soils. The soil type 1 was previously unplanted marginal soil having low organic matter, P and N contents compared to soil type 2 which was a fertile routinely legume-cultivated soil. The effect of soil fertility status was pronounced and fertile soil on average, produced 31% more nodules, 62% more biomass and 111% grain yield than marginal soil. Inoculation either with *O. ciceri* alone or its co-inoculation with *M. ciceri* produced on average higher nodules (42%), biomass (31%), grains yield (64%) and harvest index (72%) in both chickpea genotypes over non-inoculated controls in both soils. Soil 1 showed maximum relative effectiveness of Ca-34^T^ inoculation for kabuli genotypes while soil 2 showed for desi genotypes except B8/02. Desi genotype B8/02 in soil type 1 and Pb-2008 in soil type 2 showed significant yield increase as compared to respective un-inoculated controls. Across bacterial inoculation treatments, grain yield was positively correlated to growth and yield contributing parameters (*r* = 0.294^*^ to 0.838^***^ for desi and *r* = 0.388^*^ to 0.857^**^ for kabuli). PCA and CAT-PCA analyses clearly showed a site-specific response of genotype x bacterial inoculation. Furthermore, the inoculated bacterial strains were able to persist in the rhizosphere showing colonization on root and within nodules. Present study shows that plant growth promoting rhizobacteria (PGPR) inoculation should be integrated with national chickpea breading program in Pakistan especially for marginal soils. Furthermore, the study shows the potential of phytohormone producing strain Ca-34^T^ as promising candidate for development of biofertilizer alongwith nodulating strains to get sustainable yield of kabuli and desi chickpea with minimum inputs at marginal land.

## Introduction

Due to high nutritive value, chickpea (*Cicer arietinum* L.) is one of the earliest cultivated and third widely grown edible legume in tropical, sub-tropical and temperate regions of the world. Pakistan is the 2nd largest chickpea growing and third biggest chickpea producing country with an average yield of 561 kg/ha (FAO statistics, 2014^1^) where the crop is grown as the principal winter pulse crop, fodder and green manure. Crop legumes, grown in rotation with cereal crops, can improve yields of the cereals and contribute to the total nitrogen (N) pool in soil. Legumes especially chickpea occupies special position regarding nutrition as well as soil fertility and improvement. It has the ability to grow well in poor soils as well as to improve them because of its efficient N fixation system. It can happily grow on marginal, poorly fertile sandy loam land, almost exclusively under rain-fed conditions in areas of low rainfall without any chemical or biological fertilizer. Soil factor exert greater influence than bacterial inoculation on plant growth, nitrogen fixation and nutrient uptake of plant (Neumann et al., 2011).

Sustainable production depends upon the manipulation of all genetic and environmental factors that influence crops by exploiting high yielding varieties and manipulation of its symbiotic system. The plant growth promoting rhizobacteria (PGPR) induce plant's nutrient acquisition, disease tolerance and play a vital role in crop yield (Lugtenberg and Kamilova, 2009). Growth and yield of the plant have been improved by repeated inoculation of highly effective rhizobia (Hynes et al., 2008) and/or co-inoculation with PGPR (Mirza et al., 2007; Mishra et al., 2009; Tsigie et al., 2011). Improving biological nitrogen fixation (BNF) in food crops may increase plant-based protein for human consumption and increase growth of subsequent crops with lesser chemical inputs.

Implementation of PGPR-based biofertilizer technology presents economic, environmental and agronomic benefits and could be used to a larger degree to partially replace the synthetic fertilizers (Silva and Uchida, 2000; Adesemoye and Kloepper, 2009) to improve economic yield under natural conditions. Repeated incorporation of rhizobia with more effective strains coupled with the addition of “helper bacteria” can add to the BNF of the crop. Strain x variety interaction is as important as that of strain or crop variety alone (Abi-Ghanem et al., 2011). Selection of best microbial strains and plant variety/cultivar is important because a strong effect of cultivar x microbe has been reported on BNF in soybean (Israel, 1981; Israel et al., 1986), beans (Valverde and Otabbong, 1997), peanuts (Wynne et al., 1980), lentils and peas (Hafeez et al., 2000; Abi-Ghanem et al., 2011). Comparison of N_2_ fixation among various rhizobial strains demonstrated 42% variability among strains while 81% variability among different lentil cultivars (Hafeez et al., 2000). This further supports the fact that each host cultivar has a variable potential for nitrogen fixation and response toward rhizobial inoculation.

Of the two chickpea varieties, the desi type accounts for 85% and the kabuli type for 15% of the area. These two types differ in their yield potential and hence in nutrient requirements. Each ton of chickpea grain removes 121.9 kg of primary nutrients (67.3 N + 6.6 P + 48 K), 34.7 kg of secondary nutrients (18.7 Ca + 7.3 Mg + 8.7 S) alongwith ~1000 g of four micronutrients (38 Zn + 868 Fe + 70 Mn + 11.3 Cu) (Aulakh et al., 1985; Prasad et al., 2002). Chickpea is although a hard crop and can grow well even in the marginal soil and soils of varying textures. In general, the soils of chickpea-growing areas have low organic carbon content, which is indicative of low soil fertility. Chickpea can be grown successfully in soils with a pH ranging from 6 to 9. Low pH (<4.6), besides limiting some micronutrient availability, causes some toxicity problems and poor nodulation. Depending on soil fertility, climate and plant factors, nutrient, e.g., N, P, Fe, B and S deficiency in the soil causes yield losses up to 10%, 29–45%, 22–90%, 100%, and 16–30%, respectively (Ali et al., 2002).

Members of genus *Ochrobactrum* are adapted to a wide range of habitats and usually considered as free-living and ubiquitous. Although frequently isolated from the soil and rhizosphere and forming a substantial population in the rhizosphere, it has considerable effect on plant growth (Faisal and Hasnain, 2006; Príncipe et al., 2007; Chakraborty et al., 2009) and biocontrol agent (Cook et al., 1997). Furthermore, members of this genus have significant applications in the biodegradation of a range of toxic materials in soil (Yamada et al., 2008) and treatment of waste water (Ozdemir et al., 2003). *O. ciceri* was isolated from the nodules of chickpea plants in Pakistan (Imran et al., 2010) and contain IAA production ability *in vitro*. The response of inoculation and the rhizosphere colonization potential of this strain over other bacteria in the rhizosphere however, need to be determined *in vivo*.

Extensive breeding and management programs are going on for the development of high yielding chickpea genotypes with improved disease resistance but no data is available for the PGPR-inoculation response of these genotypes/varieties. This study was, therefore, planned to evaluate the response of both kabuli and desi genotypes/varieties to inoculation with PGPR to screen the best strain x genotype combination for further improvement of nodulation and yield of chickpea in local farming system. The ultimate objective was to develop a PGPR based biofertilizer for resource-poor farmers cultivating chickpea at marginal lands.

## Materials and methods

### Plant material

Six desi and four kabuli chickpea genotypes (Table 1), including both adapted varieties and advanced breeding lines (i.e., “genotypes”) were obtained from Nuclear Institute of Agriculture and Biology (NIAB), Faisalabad. The seeds were multiplied under identical conditions at a single site to minimize the seed source effect. The desi and kabuli chickpea genotypes chosen in this study represent a wide range of variation in seed size and yield, disease resistance and origin.

**Table 1:** **Description and agronomic performance of desi and kabuli chickpea genotypes**.

**Genotype**	**Type**	**100 seed weight (g)**	**Origin**	**Germination %**	**Reported Yield (Kg/Ha)**	**Blight (1–9 rating)**	***F*. wilt %**
CM2008	Kabuli	23.0 ± 0.2	NIAB^a^	91	1330	5	12
Pb-Noor-2009	Kabuli	23.1 ± 0.1	AARI^b^	75	1000	6	5
CC121/00	Kabuli	22.4 ± 0.2	NIAB	81	1430	6	8
PKV-2	Kabuli	38.2 ± 0.3	India	88	840	5	20
Pb2008	Desi	27.3 ± 0.1	AARI	87	1480	5	8
CH23/00	Desi	23.0 ± 0.4	NIAB	76	1386	4	16
B8/02	Desi	26.7 ± 0.2	NIAB	95	770	4	7
93127	Desi	21.8 ± 0.2	AARI	89	1025	3	2
CH21/02	Desi	31.8 ± 0.4	NIAB	98	1457	6	16
CM72/02	Desi	27.5 ± 0.1	NIAB	65	965	5	100

a *Nuclear Institute for Agriculture and Biology, Faisalabad (Pakistan)*.

b *Ayub Agriculture Research Institute, Faisalabad (Pakistan)*.

*F. wilt, Fusarium wilt*.

### Bacterial strains, inoculum preparation, and seed preparation

Two distinct strains of chickpea *Ochrobactrum ciceri* Ca-34^T^ (DSM 22292; CCUG 57879) and *Mesorhizobium ciceri* TAL-1148 (USDA 3100) were used for inoculation. *O. ciceri* Ca-34^T^ is an IAA producing strain and naturally resistance to ampicillin (10 μg), aztreonam (30 μg), cephradine (ß-lactams30 μg), cefixime (5 μg), amikacin (30 μg), carbenicillin (100 μg), gentamicin (10 μg), cephradine (30 μg), ceftriaxone (30 μg), paratam (105 μg), kanamycin (30 μg), rifampicin (5 μg), trimethoprim (1.25 μg)/sulfamethoxazole (23.76 μg) (25 μg), and chloramphenicol (30 μg) (Imran et al., 2010). *M. ciceri* TAL-1148 (provided by the Nitrogen Fixation by Tropical Agricultural Legumes Project, University of Hawaii, Paia, Hawaii, USA) is a reference nodulating strain being used for inoculum production worldwide. *O. ciceri* Ca-34^T^ and *M. ciceri* TAL-1148 were grown in LB and YEM, respectively, at 28 ± 2°C over-night with constant shaking. The cells were harvested with centrifugation and suspended in saline to get 10^9^ cells ml^−1^. Bacterial cultures were mixed with sterilized finely ground carrier material (finally grinded filter mud). The seeds were mixed until they become coated with a thin film of bacterial inoculum. Seeds for the un-inoculated control were coated in a similar manner with the sterilized carrier material prepared in saline. Coated seeds were air-dried in shade before sowing.

### Experimental site and design

Experiments were conducted at fields of Nuclear Institute of Biology (NIAB), Faisalabad (longitude 73°74 East, latitude 30°31.5 North, with an elevation of 184 meters above sea level). The climate of the city is extreme with very little rainfall. The soil is sandy clay loam containing 57% sand particles, 24% silt and 19% clay. Chemical properties of the soils are mentioned in Table 2. The bacterial inoculation treatments were; T1 = Ca-34^T^, T2 = TAL-1148, T3 = Ca-34^T^ +TAL-1148, T4 = Un-inoculated control. Field experiments were designed in a split-plot randomized complete block design (RCBD) with three replications at each site. The plot size was 1.0 × 1.2 m each having eight rows (3 m length), with 30/15 cm inter/intra row spacing. Two rows at the beginning and two at the end of each block were kept for protection. Genotypes (10) were used as main plot while treatments (four) were used as sub-plots. Fields were prepared by pre-sowing irrigation (10 days before field preparation) and one bag DAP (N 18%, P_2_O_5_ 46%) fertilizer @ 125 Kg/Ha applied during the field preparation. No further irrigation or fertilizer was applied during the whole experiment.

**Table 2:** **Physicochemical properties of soil (0–15 cm) from experimental fields prior to seeding**.

**Soil property**	**Soil type 1**	**Soil type 2**
Sand	57%	57%
Silt	24%	24%
Clay	19%	19%
Texture class	Sandy clay loam	Sandy clay loam
pH	7.8 ± 0.1	7.7 ± 0
Electrical conductivity (EC) d sm^−1^	1.7 ± 0.01	1.8 ± 0.02
Saturation percentage (SP)	28 ± 1	31 ± 1.2
Field capacity (%) (FC)	14 ± 0.2	15.5 ± 0.4
CO_3_ meq L^−1^	Nil	Nil
HCO_3_ meL^−1^	2.5 ± 0.3	5.2 ± 0.1
Cl meL^−1^	2.8 ± 0.0	2.9 ± 0.1
Ca+Mg meL^−1^	8.6 ± 1.2	13.0 ± 1.5
Na meL^−1^	7.4 ± 1.1	4.1 ± 1.2
K meL^−1^	0.6 ± 0.01	0.5 ± 0.03
Sodium absorption ratio (SAR)	3.6 ± 0.3	1.6 ± 0.7
Residual sodium carbonate (RSC)	nil	nil
Total N (g Kg^−1^)	0.54	0.98
Available P (mg Kg^−1^)	3.7	4.5
Organic matter (%)	0.66	0.99
Background bacterial population	3 × 10^5^	7.5 × 10^9^
Previous crops	Not vegetated for last 8 years	Regular legume (mungbean, chickpea), castor growing soil
Fertility status	Marginal	Fertile

### Measurement and data analysis

Six plants were randomly selected from the middle rows of each replicate at 60, 120, and 180 DAS (days after sowing). Plant height and fresh weights were recorded immediately while dry weights were recorded after oven drying at 65°C for 48 h. Number of seeds per pod was calculated by counting 25 randomly selected pods/plant from each treatment and taking their averages. 100-seed weight was calculated by taking the average of 300 seeds from each treatment. Grain and straw yield was calculated by taking the yield of 25 random plants from each treatment and then converted to Kg/ha. Total biomass was calculated from weight of sun-dried 25 plants (including grain and straw) from each treatment and converted to Kg/ha. Harvest index (HI) percentage was calculated using the formula: HI (%) = Seed yield/total biomass × 100. Relative effectiveness (RE) was determined by following equation and expressed in percent as described by Maâtallah et al. (2002).

$$\begin{array}{l} \text{Relative effectiveness }\left(\text{RE}\right)=\left(\text{D}{\text{W}}_{\text{ino}}∕\text{D}{\text{W}}_{\text{cont}}\right)\times 100 \end{array}$$

Where DW_ino_ = Dry weight of inoculated plant; DW_cont_ = dry weight of un-inoculated control plants. Split-plot ANOVA was performed using Statistix 8.1 and the means [±standard deviation (SD)] were compared using Tukey HSD all-pairwise comparison test. Mean separation was done using LSD at *p* = 0.05. Regression and Principal Component Analysis was performed using SPSS software package version 17.0 (SPSS, Inc., Chicago, IL).

### Detection of inoculants

Bacteria were quantified from chickpea roots at 30 DAS and nodules at 80 DAS using serial dilution plating technique. The nodules and roots were carefully detached from the plants. One gram soil tightly adhering to the roots was used to prepare serial dilution. Nodules were washed thoroughly with sterile distilled water, then flame sterilized by dipping in ethanol. Sterilized nodules were incubated overnight on LB agar plates to see the surface contamination. Only those nodules were processed for serial dilutions that did not show any surface contamination. The dilutions were spread onto YEM-congored to recover TAL-1148. Ca-34^T^ was recovered on YEM-congored plates containing kanamycin (30 μg), aztreonam (30 μg) and rifampicin (5 μg). Colonies showing similar colony morphologies to those of the inoculant strains were subjected to immunoblotting. Polyclonal antibodies were raised in 6 months old female albino rabbits and immunoblotting was carried out using standard protocols (Somasegaran and Hoben, 1994). Both the antisera showed no reactivity with antigens of *Pseudomonas* spp. 96-51 (Rasul et al., 1998) and MST4.1 (Hafeez et al., 2006), *Meso/rhizobium* spp. IC-94 and IC-2002 (*ICRISAT India; Provided by Solange Oliveira, Departamento de Biologia, Universidade de Âvora, Portugal), Bradyrhizobium* spp. TAL-102 (Provided by NIFTAL), MN-S and *Agrobacterium tumefaciens*Ca-18 (Hameed et al., 2004), *Azospirillum lipoferum* JCM-1270; DSM1842 and *A. brasilense* JCM-1224^T^; DSM1690 (Provided by Japan Collection of Microorganisms) and *Ochrobactrum* spp. (provided by DSMZ, Germany) including *O. tritici* spp. LAIII 106 and SCII24^T^, *O. gallinifaecis* ISO 196^T^, *O. oryzae* MTCC 4195^T^, *O. lupini* LUP-21^T^. A very weak signal was observed for the cross-reactivity of TAL-1148 with IC-94 and Ca-34^T^ with LAIII 106 but the signal intensity was very low compared to the positive reaction hence false detection was unlikely.

*Ochrobactrum*-like colonies were further confirmed by colony PCR using *O. intermedium* specific forward primer F4 (Romero et al., 1995) and Ca-34^T^ specific reverse primer OcA-34 5′ -GCCCCCCTTTAAAATTTCAG-3′. This primer was designed against the previously reported 46 nucleotides insertion in 16S *rRNA* of Ca-34^T^ (Imran et al., 2010). This 46bp insertion is present at *E. coli* 16S *rRNA* position 187 that folds into a stem loop structure and reported to prolong the helix H184 when placed on the 16S *rRNA* gene sequence secondary structure. The nodules were crushed on Fast Prep instrument in 200 μL distilled water and nodule lysates were used for PCR-detection of Ca-34^T^ using F4 and OcA-34. Moreover, the strain identity was confirmed using random primer OPC-13 that generates a specific fingerprint for Ca-34^T^. Preparation of PCR reaction and amplification conditions were same as described (Imran et al., 2010).

## Results

### Effect of inoculation on chickpea nodulation

**Figure 1:**
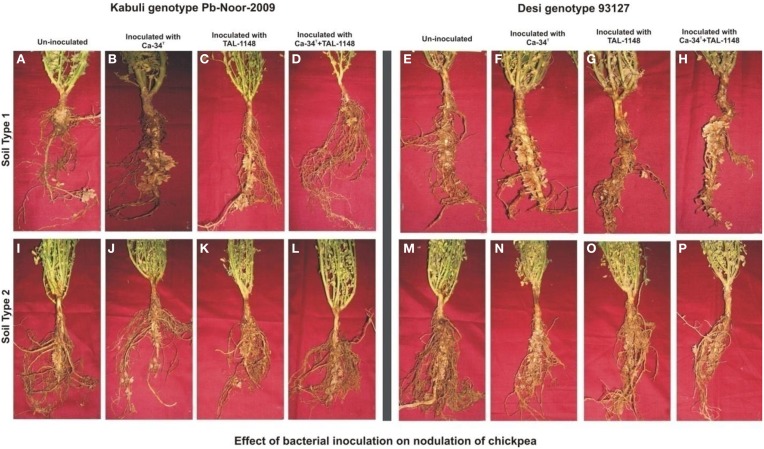
**Effect of bacterial inoculation on nodulation of chickpea genotypes in different soils**. The nodules induced after inoculation on kabuli genotype Pb-Noor-2009 in marginal **(B–D)** and fertile soil **(J–L)** as compared to respective non-inoculated control plants **(A,I)** and Desi genotype 93127 in marginal **(F–H)** and fertile soil **(N–P)** as compared to respective non-inoculated controls **(E,M)**.

Nodulation exhibited no consistent pattern with plant genotype, inoculation or soil type. Nodules were mostly observed along entire primary/tap root in most of the genotypes forming crown (whorls) around the root and very few nodules were observed on secondary roots (Figure 1). Nodule number and nodule biomass showed differential genotype response toward inoculation in both soils (*P* < 0.05) and generally nodule number and biomass was higher in soil 2 (Table 3). Kabuli genotypes produced higher nodules and nodule biomass in unplanted soil than desi genotypes. Nodulation in kabuli genotypes Pb-Noor-2009, PKV-2 and desi genotypes Pb-2008, CH23/00 was significantly increased either by single inoculation with Ca-34^T^ (up to 42%) or co-inoculated along-with TAL-1148 (up to 29%) in both soils. Inoculation with TAL-1148 alone however, had non-significant effect on nodulation of most of the kabuli and desi genotypes in any soil. Comparison of treatment means (averaged over genotypes and replicates) showed that in soil 1, nodulation in Ca-34^T^-inoculated plants was maximum (55.85 nodules per plant) followed by its co-inoculation (50.93 nodules per plant) while in soil 2, nodulation was maximum in co-inoculated plants (72.15 nodules per plant) followed by Ca-34^T^-inoculated plants (62.23 nodules per plant).

**Table 3:** **Effect of inoculation on chickpea symbiotic performace in different soils**.

**Varieties**	**Treatments**	**Nodule number**		**Nodule dry weight (g)**	
		**Soil 1**	**Soil 2**	**Soil 1**	**Soil 2**
**DESI VARIETIES**					
Pb-2008	Ca-34	58.75±1.2	77.25±4.5	0.315±0.14	0.495±0.01
	TAL-1148	30.5±2	45.75±0.6	0.232±0.01	0.422±0.05
	Mix	31.5±0.5	138.5±3.4	0.385±0.11	0.827±0.14
	Control	42.25±2	52.25±1	0.375±0.04	0.625±0.09
CH23/00	Ca-34	50.25±1.1	99.75±4.6	0.307±0.03	0.644±0.01
	TAL-1148	21.5±0.5	66.5±2.6	0.167±0.04	0.784±0.08
	Mix	54.5±2	65.75±3.4	0.495±0.11	0.665±0.03
	Control	26±1	63.25±2.2	0.192±0.01	0.625±0.11
B8/02	Ca-34	50.5±2	72.5±3.5	0.367±0.06	0.447±0.12
	TAL-1148	27.75±1	71.25±6	0.137±0.01	0.842±0.11
	Mix	58.25±1	87±4.5	0.242±0.03	0.572±0.15
	Control	54±4	69±4	0.662±0.02	0.784±0.14
93127	Ca-34	41.2±2.5	63.3±5	0.265±0.01	0.555±0.13
	TAL-1148	39.25±0.6	54.75±3	0.287±0.03	0.395±0.02
	Mix	79±2.6	56.25±3.3	0.647±0.02	0.297±0.04
	Control	45.5±3.4	54.25±4	0.212±0.01	0.4±0.04
CH21/02	Ca-34	72.25±0.4	59±0.75	0.312±0.00	0.55±0.05
	TAL-1148	67.5±0.1	58.25±1.5	0.452±0.04	0.50±0.02
	Mix	56.41±2	58±1.6	0.312±0.02	0.52±0.01
	Control	61±2	47.75±4	0.392±0.01	0.487±0.01
CM72/02	Ca-34	48.7±1.5	52.25±2.6	0.23±0.06	0.322±0.02
	TAL-1148	56.75±2	35.8±4	0.305±0.01	0.20±0.00
	Mix	47.75±3	33.75±4	0.092±0.01	0.245±0.01
	Control	31.25±0.8	44.25±1.9	0.132±0.02	0.302±0.02
**KABULI VARIETIES**					
CM-2008	Ca-34	65.75±2	74.5±2	0.62±0.03	0.827±0.13
	TAL-1148	54±1.5	46.5±3	0.50±0.01	0.617±0.11
	Mix	40.2±5	66.75±2.5	0.345±0.01	0.411±0.01
	Control	34.7±4	56.25±1.8	0.222±0.04	0.71±0.09
Pb-Noor-2009	Ca-34	99.5±2.1	51.5±1.6	0.427±0.02	0.455±0.01
	TAL-1148	31.5±2.5	49.25±1.4	0.217±0.02	0.477±0.03
	Mix	96.75±3	60.25±3.2	0.815±0.07	0.31±0.02
	Control	29.25±2	34±4	0.207±0.05	0.30±0.02
CC121/00	Ca-34	30.25±3	66±2.1	0.165±0.03	0.57±0.07
	TAL-1148	47.75±3.1	31±2	0.322±0.01	0.275±0.01
	Mix	35.25±3.3	71.7±3	0.25±0.01	0.565±0.06
	Control	36.25±4	64.5±3.3	0.26±0.01	0.74±0.04
PKV-2	Ca-34	61.25±0.7	72.5±3	0.255±0.02	0.587±0.04
	TAL-1148	40.25±4	50.25±2	0.122±0.02	0.485±0.08
	Mix	66.75±4.5	90.5±2.4	0.29±0.04	0.835±0.04
	Control	22.75±2.8	62.25±4.3	0.102±0.01	0.52±0.02

*± Shows the standard error of three replicates*.

### Effect of inoculation on plant biomass, relative effectiveness and crop yield

Genotype (G) response was highly significant (*P* < 0.05) at all harvests of 60, 80, and 120 DAS. Inoculation (T) response was significant (*P* < 0.05) at 60 and 80 DAS only. Kabuli genotypes produced higher shoot and root lengths while desi genotypes produced higher root and shoot weights irrespective of soil condition (see Supplementary Tables 1, 2 for detail). In soil 1, the plants inoculated either with Ca-34^T^ or co-inoculated along-with the nodulating strain produced taller plants, higher root and plant biomass, more primary and secondary branches, straw weight and grain yield as compared to inoculation with TAL-1148 alone. In soil 2 (fertile soil), inoculation with TAL-1148 showed better results as compared to other treatments. When treatment means were compared (averaged over genotypes), effect of inoculation on chickpea plant height, primary/secondary branches was non-significant in soil 2 while statistically significant in soil 1 showing maximum inoculation response in co-inoculated plants. Yield data of individual plant showed a significant genotype-dependent inoculation response (*P* < 0.05) in both soils. Desi genotypes Pb-2008, CH23/00, and CH21/02 produced more primary and secondary branches, straw weight, grain yield, and seeds per plant in both soils. Generally, co-inoculation of Ca-34^T^+TAL-1148 significantly improved yield contributing parameters in marginal soil (soil type 1) whereas the response of inoculation in fertile soil (type 2) was, although higher than soil 1, non-significant as compare to uninoculated control. Inoculation with Ca-34^T^ resulted in 63 and 22% increase while its co-inoculation with TAL-1148 resulted in 54 and 15% increase in grain yield (Kg/Ha) in marginal and fertile soils, respectively over control.

RE of inoculation (Table 4) was highest for desi variety B8/02 (321.69) inoculated with Ca-34^T^+TAL-1148 followed by kabuli genotype CM2008 (301.43) inoculated with Ca-34^T^. Over all, kabuli genotypes showed higher RE as compared to desi genotypes in both soils while RE of soil 1 was higher as compared to soil 2.

**Table 4:** **100-seed weight, relative effectiveness and harvest index of desi and kabuli chickpea genotypes after inoculation with PGPR in different soils**.

**Variety**	**Treatment**	**100-grain weight (g)**		**Grain yield (Kg/ha)**		**Relative effectiveness(%)**		**Harvest index (%)**	
		**Soil 1**	**Soil 2**	**Soil 1**	**Soil 2**	**Soil 1**	**Soil 2**	**Soil 1**	**Soil 2**
**DESI VARIETIES**									
Pb-2008	Ca-34	27.38 (1.2)	33.47 (0.4)	1857 (93)bcde	5898.5 (65)a	88.31	82.63	33.07	73.75
	TAL-1148	18.3 (0.8)	29.24 (0.5)	1821.5 (54)bcde	5153.7 (95)ab	82.17	122.65	38.16	62.73
	Mix	26.02 (0.45)	30.29 (0.1)	2274 (66)abcde	4592 (101)ab	110.94	128.09	35.39	56.91
	Control	24.92 (0.7)	27.58 (0.7)	2152 (78)abcde	3793.98 (54)abc	–	–	33.36	54.87
CH23/00	Ca-34	25.56 (0.8)	28.11 (0.3)	750.4 (12)de	4098 (58)abc	74.49	125.96	20.62	50.31
	TAL-1148	22.26 (1.14)	25.9 (0.3)	848 (19)de	3480.96 (69)abc	84.09	95.93	19.03	52.67
	Mix	23.83 (0.6)	28.4 (0.6)	1247.5 (76)de	4047.8 (99)abc	142.29	98.41	18.82	55.98
	Control	20.94 (0.4)	26.97 (0.4)	1509.6 (59)bcde	3901.65 (53)abc	–	–	25.32	50.67
B8/02	Ca-34	26.35 (1.1)	27.49 (0.7)	3729.6 (77)ab	3797 (114)abc	279.56	74.58	59.86	61.83
	TAL-1148	31.26 (0.3)	28.89 (0.1)	2762.8 (65)abcd	3731.8 (25)abc	321.69	138.44	47.30	45.57
	Mix	20.34 (0.5)	29.39 (0.1)	4270 (54)a	4724 (22)ab	372.94	65.22	50.86	65.97
	Control	29.41 (0.6)	23.43 (0.5)	1152 (45)de	4355.6 (59)ab	–	–	51.03	57.85
93127	Ca-34	25.3 (0.4)	26.3 (0.4)	2186.7 (20)abcde	3382 (63)abc	86.79	92.78	57.43	61.72
	TAL-1148	22.31 (0.2)	31.24 (0.4)	2010 (48)abcde	3432 (22)abc	127.18	99.59	41.64	56.52
	Mix	25.09 (0.1)	28.95 (0.7)	2050.2 (54)abcde	4724 (36)ab	130.89	142.23	41.30	59.10
	Control	24.54 (0.6)	25.8 (0.1)	1827 (42)bcde	3858 (38)abc	–	–	52.23	62.36
CH21/02	Ca-34	27.55 (1.2)	34.38 (1.1)	2426.5 (24)abcde	4865 (52)ab	34.61	77.05	63.82	58.89
	TAL-1148	32.42 (0.4)	36.64 (0.1)	2308.8 (82)abcde	3908 (54)abc	62.166	71.31	50.88	52.95
	Mix	31.46 (0.4)	31.81 (0.2)	2445.9 (33)abcde	4616.5 (45)ab	49.72	52.95	61.32	65.53
	Control	30.35 (0.6)	30.43 (0.4)	2147.9 (25)abcde	4279 (11)ab	–	–	42.01	45.71
CM72/02	Ca-34	31 (1)	32.15 (0.5)	1308.7 (38)cde	4711.95 (22)ab	75.78	103.14	57.07	58.06
	TAL-1148	32.14 (0.5)	32.68 (0.4)	1668.6 (40)bcde	3812.9 (47)abc	111.64	75.11	55.77	62.24
	Mix	31.89 (0.4)	32.14 (0.4)	1457 (25)bcde	4303.5 (33)ab	163.42	90.60	44.75	59.26
	Control	32.34 (0.1)	30.9 (0.3)	1091 (52)de	4708.6 (24)ab	–	–	43.38	60.34
**KABULI VARIETIES**									
CM-2008	Ca-34	22.66 (0.1)	27.7 (0.1)	3622.5 (62)abc	3473 (36)abc	301.43	273.78	40.32	55.68
	TAL-1148	18.03 (0.2)	27.39 (0.2)	1478.5 (24)bcde	2934.8 (44)abc	132.62	258.08	30.27	46.80
	Mix	18.25 (0.4)	27.1 (0.1)	1430.8 (43)bcde	3392.7 (25)bc	125.64	235.67	33.43	40.44
	Control	26.94 (0.6)	25 (0.1)	303 (33)e	951 (22)c	–	–	16.22	32.29
Pb-Noor-2009	Ca-34	27.74 (0.1)	26.7 (0.3)	2025.8 (32)abcde	3951 (51)abc	133.90	71.66	38.64	76.45
	TAL-1148	28.69 (0.2)	22.69 (0.5)	1857 (38)bcde	3439.9 (84)abc	123.45	208.60	38.56	50.60
	Mix	27.52 (0.2)	26.04 (0.1)	1944.7 (41)bcde	3480.9 (26)abc	166.29	137.66	29.49	62.83
	Control	28.44 (0.2)	22.44 (0.5)	1133 (42)de	3313.5 (23)abc	–	–	33.37	67.05
CC121/00	Ca-34	21.67 (0.1)	25.84 (0.5)	2241 (21)abcde	3820.6 (22)abc	193.46	106.11	54.14	68.04
	TAL-1148	22.92 (0.5)	24.07 (0.6)	2264 (60)abcde	2965.9 (29)abc	234.85	126.95	52.10	48.22
	Mix	25.13 (0.4)	27.48 (1.6)	2399.8 (22)abcde	4368.9 (61)ab	272.81	152.89	48.62	53.99
	Control	17.76 (0.8)	25.96 (1.5)	1172 (55)de	3181 (62)abc	–	–	44.35	52.83
PKV-2	Ca-34	33.58 (0.2)	39.62 (0.2)	1431.9 (32)bcde	4659.8 (32)ab	134.41	132.84	43.54	67.08
	TAL-1148	29 (1)	38.42 (0.2)	815.9 (41)de	3411 (18)abc	81.472	133.52	35.09	61.07
	Mix	30 (0.5)	40.29 (0.3)	890 (22)de	3064 (65)abc	84.49	146.53	40.58	55.61
	Control	30 (0.5)	34.64 (0.4)	688 (54)de	2440.9 (32)bc	–	–	31.53	57.37

*Means in each column followed by the same letter do not differ significanlty at α 0.05(LSD). The standard error of replicates is presented in paranthesis*.

Overall, harvest index (HI) was genotype-dependent and generally higher in fertile soil (type 2) for both genotypes (Table 4). Most of the genotypes inoculated with Ca-34^T^ showed increase in HI in both soils (up to 148%).

Effect of inoculation on seed size (100 grain weight) was genotype-dependent and maximum in fertile soil (type 2; Table 4). Seed size was highest in desi genotypes PKV-2, Pb-2008 and CH23/00 in plants inoculated with Ca-34^T^ or co-inoculated with TAL-1148. The desi genotypes 93127 and CH21/02 showed improved seed size with TAL-1148 inoculation as compared to other treatments.

Grain yield was found to be significantly affected by inoculation in both soils (Table 4). The yield was maximum for mix-inoculated plants of desi variety B8/02 (4270 Kg/Ha) in soil 1 and Ca-34^T^-inoculated plants of desi variety Pb-2008 (5898 kg/Ha) in soil 2. Grain yield of kabuli genotypes were least affected by soil type or inoculation treatment. Comparison of treatment means (Table 5) showed that yield was maximum in Ca-34^T^-inoculated plants in both soils.

**Table 5:** **Tukey HSD all-pairwise comparisons test for treatments (***averaged over genotypes***) and genotypes (***averaged over treatments***) calculated for biomass and grain yield in two soils**.

	**Biomass (Kg/ha)**		**Grain yield (Kg/ha)**	
	**Soil 1**	**Soil 2**	**Soil 1**	**Soil 2**
**TREATMENTS** ***(AVEREGED OVER GENOTYPES)***				
Ca-34	4674.1a	6841.3a	2158.0a	4265.8a
TAL-1148	4331.9ab	6740.3a	1783.5ab	3791.1a
Mix	5161.6a	6929.1a	2041.1a	3991.1a
Control	3570.7b	6363.2a	1317.7b	3478.4a
Standard error for comparison	200.76	245.77	110.40	170.95
**GENOTYPES** ***(AVEREGED OVER TREATMENTS)***				
Pb-2008	5796.0a	7757.8a	2026.3bc	4859.6a
CH23/00	5238.0ab	7473.8a	1088.9d	3888.9ab
B8/02	5690.7a	7209.2ab	2978.7a	4152.2ab
93127	4285.2bc	6328.7abc	2018.5bc	3766.5ab
CH21/02	4313.2bc	8045.4a	2332.2ab	4417.2ab
CM72/02	2756.8de	7309.9ab	1381.4cd	4384.2ab
CM-2008	4851.0abc	5252.8c	1708.7bcd	2413.0c
Pb-Noor-2009	5019.3abc	5622.1bc	1740.2bcd	3546.3bc
CC121/00	3900.1cd	6561.1abc	2019.4bc	3584.2bc
PKV-2	2495.6e	5624.0bc	956.5d	3394.0bc
Standard error for comparison	348.87	529.88	274.48	363.72

*Means in the same column followed by the same letter do not differ significantly at α 0.05(LSD)*.

Comparison of treatment means and genotype means (Table 5) showed that biomass was lower in soil 1 (4331–5161 kg/Ha) as compared to soil 2 (6363–6929 kg/Ha). Maximum biomass was obtained in plants with mix inoculation in both soils. Of genotypes, desi types produced more biomass as compared to kabuli types (Table 5).

### Relationship among parameters

The whole data was subjected for correlation analysis using SPSS and a positive linear relationship was found (*r* = 0.26–0.856^**^) between grain yield and measured plant growth and yield parameters. Kabuli genotypes specifically showed higher correlation coefficient ratio (*r*-values) but no significant trait correlation was observed in any genotype after bacterial inoculation.

**Figure 2:**
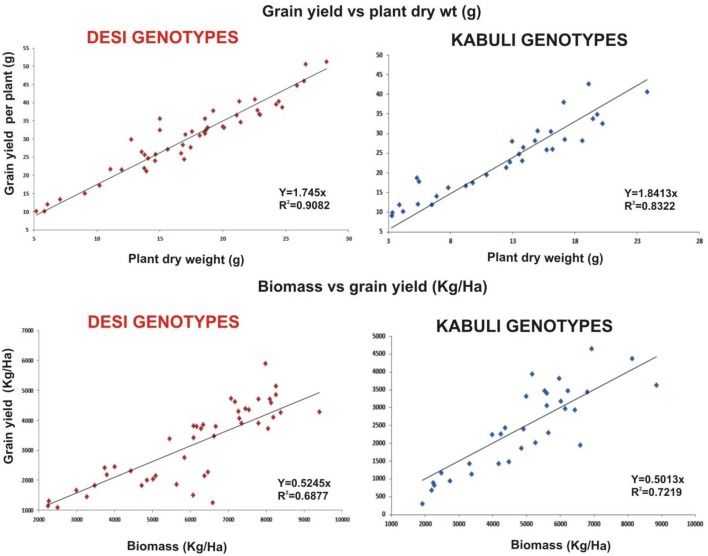
**Grain yield response to plant dry weight and biomass as a function of bacterial inoculation in desi and kabuli chickpea genotypes in two soils**. The data from two soils and four treatments has been jointly loaded on graph to evaluate the response of genotypes. Graph shows a positive linear relationship of plant dry weight to grain yield per plant and plant biomass to grain yield in both genotypes with significnalty higher *R*^2^-values.

**Figure 3:**
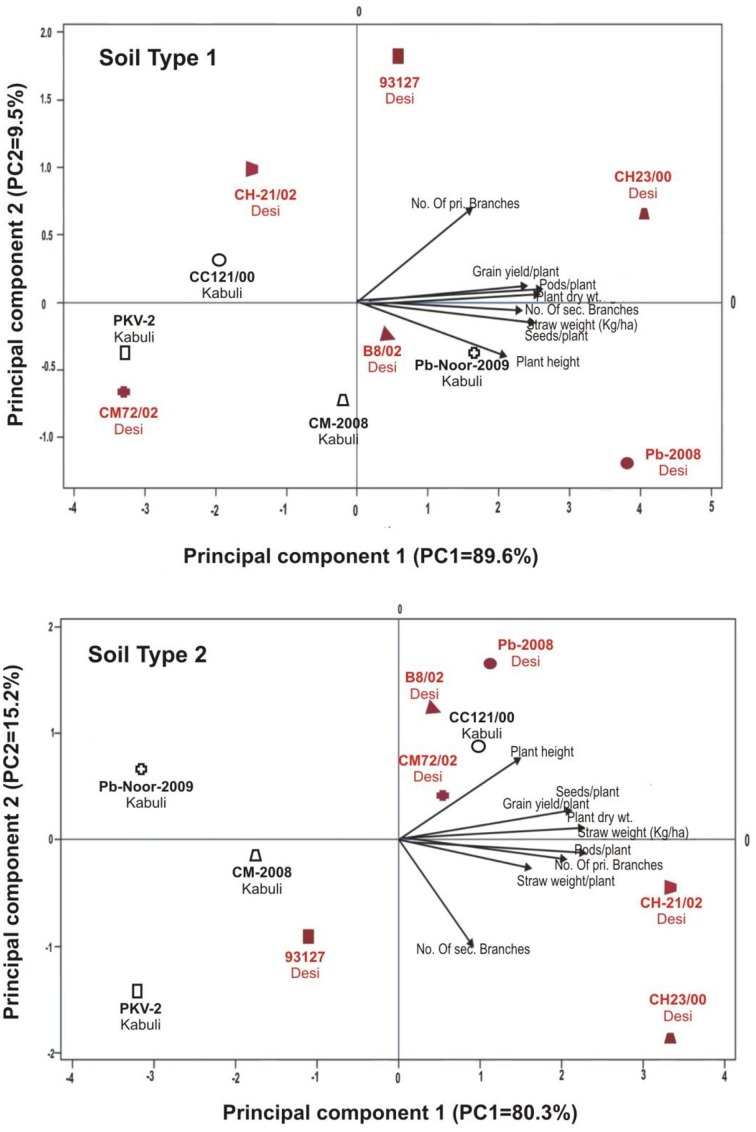
**Categorical Principal component analysis (CATPCA) of plant traits measured across desi and kabuli chickpea genotypes in two different soils ***(Total variance explaiened: 99% for soil 1, 95% for soil 2)*****. CATPCA is a non-linear PCA. Factor loadings in PC1 and PC2 are presented as vectors using external scale.

**Figure 4:**
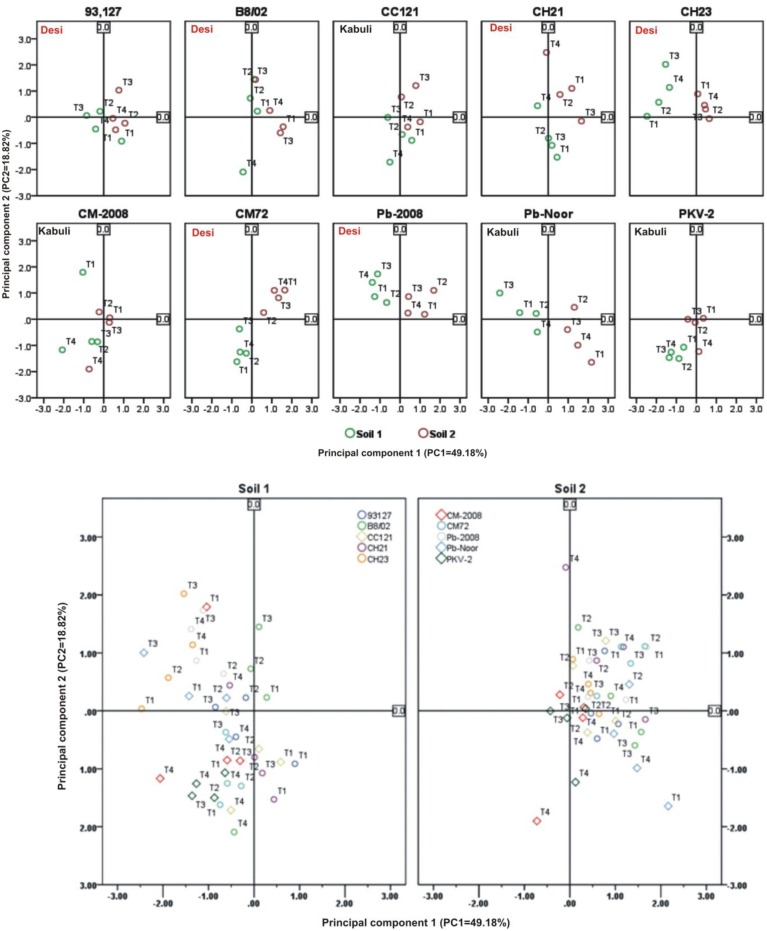
**Principal component analysis (PCA) showing the response of chickpea genotypes after four bacterial inoculation treatments in two different soils; Loaded as genotypes (upper panel) and soil type (lower panel) ***(Total variance explaiened: 68%)*****. T1 = Ca-34^T^, T2 = TAL-1148, T3 = Ca-34^T^+TAL-1148, T4 = non-inoculated Control.

Linear regression effectively modeled the positive relationship of grain yield with plant dry weight, nodule biomass and straw yield, accounting for 70–82% of total variance. A positive quadric regression (*R*^2^ = 0.916; 0.908) was observed for nodule parameters with plant dry weight and grain yield and for harvest index with plant biomass and nodule number against genotype, inoculation and soil type accounting for more than 80% of the total variance. When modeled separately, a positive regression was observed (Figure 2) for different parameters in both kabuli and desi genotypes. CAT-PCA (Figure 3) and PCA (Figure 4) captured more than 70–95% of the variance and clearly demonstrated the key genotype difference in both soils. The effect of soil was more pronounced showing all the genotypes in marginal soil loaded on negative while in fertile soil loaded on positive quadrant (Figure 3). PCA showed that soil effect was pronounced and most of the chickpea growth parameters were strongly positively correlated to each other (*r*^2^ < 0.99) and positively loaded on PC1 (Figure 4).

### Detection of inoculants

**Figure 5:**
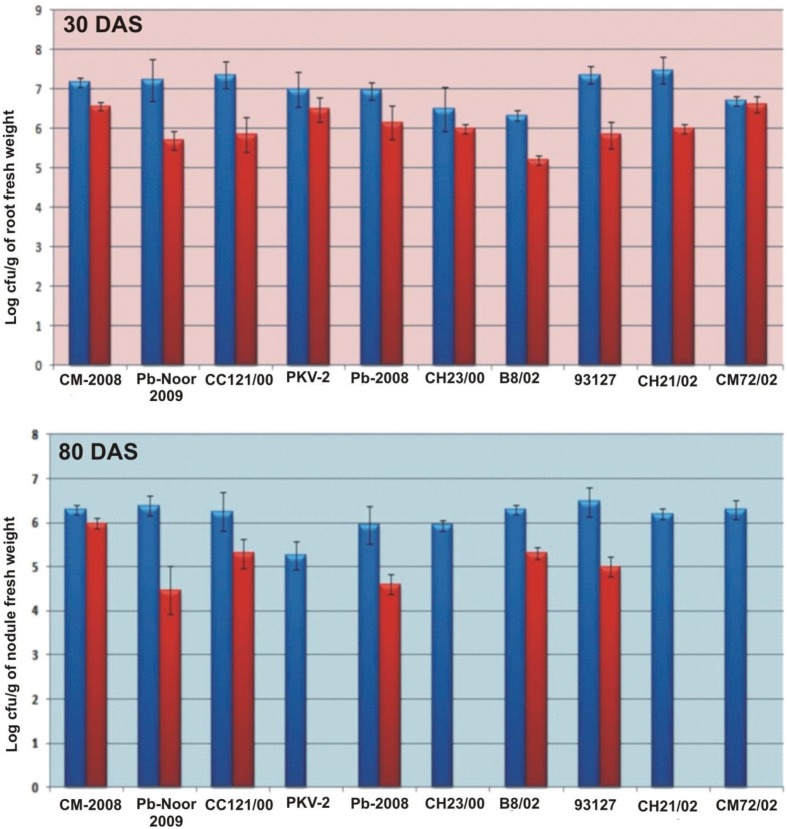
**Bacterial population (log of cfu) from roots and nodules of 10 chickpea genotypes inoculated with Ca-34^T^ in soil 1**. (The blue bars represent the total bacterial population obtained on LB while red bars indicate the Ochrobactrum-like population on YEM-congored agar containing antibiotic).

**Figure 6:**
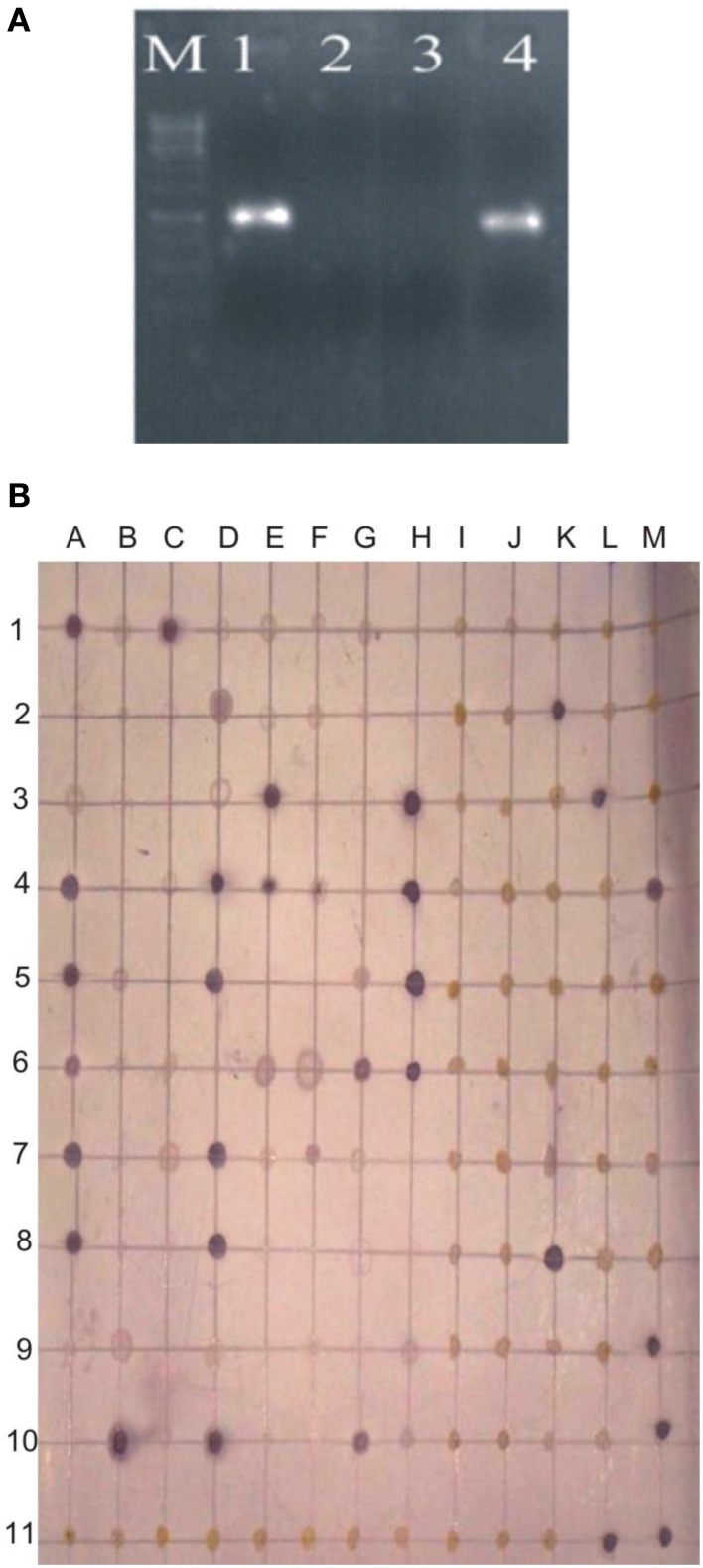
**(A)** PCR-based detection and confirmation of *Ochrobactrum ciceri* Ca-34^T^ colonies recovered from nodules of chickpea using specific primers. M, DNA marker; Lanes 1,2, Two nodule isolates obtained from chickpea plants (kabuli genotype CM-2008) inoculated with Ca-34^T^; 3, negative contorl; 4, Positive control (Ca-34^T^ pure culture). **(B)** Representative immunoblot of nodule and root isolates. The picture shows the image of the nitrocellulose membrane hybridized with the antibodies of Ca-34^T^. Columns A-M antigens loaded to the membrane; Lane 1–3, antigens prepared from nodule isolates; Lane 4–6, crushed nodules (nodule lysates); Lane 7–10, antigens prepared from root isolates; A11-K-11, negative controls including water and other *Ochrobactrum* spp. strains mentioned in methodology; L11 and M11, Ca-34^T^ (positive control).

Survival of inoculated bacteria within rhizosphere and nodule of chickpea and their persistence throughout the crop growth was tested. Based on plate-assay, it was observed that of total bacterial population attached to chickpea roots, 1.5% was similar to *Ochrobactrum*. In nodules, its population ranged from 1 × 10^3^ to 8 × 10^5^ per gram of nodule fresh weight (Figure 5) constituting about 0.04–5% of total cultureable nodule population. These results were confirmed by specific PCR (Figure 6A) immunoblotting (Figure 6B), and randomly amplified polymorphic DNA (RAPD) analysis with primer OPC-13. Although, different genotypes showed variable response in both soils but kabuli genotypes showed more colonization in nodules as compared to desi genotypes. The presence of TAL-1148 was confirmed only in nodules and the population ranged from 5 × 10^4^ to 8 × 10^7^ in different genotypes.

## Discussion

Pakistan ranks the third in terms of chickpea production globally, where, the Thal desert that cannot support/sustain major cash crops due to low fertility and lack of artificial irrigation is well known as home of chickpea. The cultivation in nutrient-deficient soils coupled with inadequate or no crop management (fertilizer, moisture application) results in low yield of crop (Kantar et al., 2007). High yield and disease resistance must be an essential traits for breeding (Micke, 1993; Richards, 2000) but needs to be combined with traits that improve crop economics such as increased N_2_ fixation capacity (Herridge and Rose, 2000). The chickpea breeding program is also hampered by the low fertility status of soil on which the crop is usually grown. Rhizobial inoculation to legumes especially chickpea exert positive effects on growth and yield (Rodríguez-Navarro et al., 2000; Kyei-Boahen et al., 2005) in soils containing in-effective rhizobia (Sharma et al., 1983; Beck et al., 1991). The success of inoculation, however, depends on the environmental conditions, soil richness (Bottomley, 1992; Graham, 1992), number and application method of effective rhizobial cells (Brockwell and Bottomley, 1995; Brockwell et al., 1995), presence of high population of competing strains of rhizobia (Thies et al., 1991) and plant genotype (Hafeez et al., 1998). This study has evaluated and demonstrated the likely contribution of PGPR inoculation toward growth promotion of kabuli and desi chickpea under different soil conditions.

Both desi and kabuli genotypes are botanically similar but genotypically different. They represent a wide range of variation in seed size and origin (parent source). Furthermore, the soil where these genotypes were evaluated, contrasts in nutrient and fertility status. One was an unplanted, marginal soil where no crop was grown for 8 years (soil type 1). Background total bacterial population of this soil was 3 × 10^5^. The other was fertile, regular legume growing soil where legumes (mungbean, chickpea) and castor were routinely grown (soil type 2) having bacterial population 7.5 × 10^9^. The experiments were conducted with a very little addition of external fertilizer (added before the experiment) and with zero irrigation. As chickpea can perform well under conditions of moisture stress in marginal soils, hence, drought tolerance in this crop is extremely desirable attribute for moisture deficient areas of the country. The inoculation response was more significant in soil 1 having poor indigenous rhizobial population and fertility (can be categorized as marginal land) which shows that soil fertility, nutrient status, and indigenous population of bacteria have a vital role in the development of plant microbe interaction. The pronounced effect of soil was very evident in PCA analysis where both soil types loaded differently on PC1 and PC2. The evidences suggest that this interaction is mainly controlled by indigenous microbial population, soil richness and by plant genotype hence, their co-selection under a given set of soil and environmental conditions may enhance the amount of fixed N. The inoculation effect was different in both soils which show that soil fertility, nutrient status and indigenous population of bacteria have a vital role in the development of plant microbe interaction. In fertile soil, the average genotype yield in un-inoculated control plants was higher than the reported yield (Table 1). This high yield might be due to the *Meso/brady/Rhizobium*-rich soil and excellent fertility status of the soil. It is believed that general/universal inoculum for all systems is impossible to develop as effectiveness depends upon plant type, soil type, weather conditions and many unidentified factors (Adesemoye et al., 2009). The data showed that soil type 1 was better for growing kabuli genotypes while soil type 2 was better for cultivation of both genotypes.

Inoculation of nodulating reference strain TAL-1148 alone showed non-significant nodulation response in both soils. High background rhizobial population usually hampers the growth, survival and colonization of inoculating rhizobial strains in the soil. Where indigenous population offers more competition, the effect of inoculation usually is non-significant. Desi genotype Pb-2008 showed increased nodulation in plants co-inoculated with Ca-34^T^+ TAL-1148. It is already established that inoculation with nitrogen-fixing/nodulating strain combined with PGPR exert more beneficial effect on plant as compared to single inoculation (Hameed et al., 2004). The synergistic effect of co-inoculating strains is well established in chickpea (Mirza et al., 2007; Adesemoye and Kloepper, 2009). Increased biomass and grain yield in Ca-34^T^+ TAL-1148 co-inoculated plants may be attributed to the nitrogen fixation potential of TAL-1148.

Overall Ca-34^T^-inoculation significantly increased nodule number, plant height, root proliferation and biomass, primary and secondary branching, plant biomass, straw weight and grain yield in single as well as multi-strain combination treatment. Most responsive genotypes toward Ca-34^T^-inoculation were all four kabuli genotypes and two desi genotypes B8/02 and Pb-2008. These genotypes showed maximum increase in nodulation, biomass and grain yield by Ca-34^T^-inoculation. Varietal difference for harvest index has been reported in chickpea, mungbean (Singh et al., 1980; Malik et al., 1981) and rice (Fida et al., 1993). The importance of changes in dry weight partitioning between organs have focused attention of scientists on harvest index as a specific selection criterion for plant breeders as the productivity of grain crops depends not only on dry matter accumulation (Kumar et al., 2006), but also on its effective partitioning to economically important plant parts. Improved harvest index has been responsible for the grain yield potential increase among major cereal species (Frey, 1981).

The root proliferation coupled with increased nutrient uptake and yield of plant might be the result of the production of IAA by *O. ciceri* strain Ca-34^T^. IAA is a plant growth hormone that enhances the lateral root development in plants to facilitate uptake of more nutrients and water, consequently improving growth and yield (Barazani and Friedman, 1999; Shahid et al., 2012; Ali et al., 2014). Apart from IAA, *Ochrobactrum* spp. strains are involved in nutrient mobilization (phosphate and zinc), AHL-production (Imran et al., 2014), production of siderophores (Chakraborty et al., 2009), antibiotic 2, 4-DAPG (Hassan et al., 2010) that directly or indirectly promote plant growth. Positive effects of inoculation with *Ochrobactrum* spp. have been reported in maize (Príncipe et al., 2007), mungbean (Faisal and Hasnain, 2006), wild *Coffea Arabica* L. (Muleta et al., 2008) under normal soils, and in chickpea in chromium contaminated soils (Riaz et al., 2010).

*Ochrobactrum* species, usually considered as free-living, are adapted to a wide range of habitats including soil, rhizosphere (Lebuhn et al., 2000), cotton root interior (McInroy and Kloepper, 1994), wheat roots (Sato and Jiang, 1996), deep-water rice endophyte (Verma et al., 2004; Tripathi et al., 2006) and nodules of *Acacia mangium* (Ngom et al., 2004), *Lupinus albus* (Trujillo et al., 2005), *Cytisus scoparius* (Zurdo-Pineiro et al., 2007) and chickpea (Imran et al., 2010). Although omnipresent, *Ochrobactrum* species are considered as weak colonizers of plants and less competent in the rhizosphere. Persistence of the both inoculating strains in the rhizosphere and nodules of both kabuli and desi genotypes, specifically *O. ciceri* Ca-34^T^ within the nodules, shows that they are competitive and can maintain substantial population level when inoculated to the seeds. As the population was obtained on antibiotic plates which were later confirmed by immunoblotting, specific PCR and RAPD, so the possibility of getting contaminants were ruled out. Our findings are in line with the earlier findings (Lebuhn et al., 2000, 2006) reporting that *Ochrobactrum* species constitute 2% of total rhizosphere and 0.3% of rhizoplane population.

As described in this study, several others reported a strong influence of plant variety on N_2_ fixation. To enhance the amount of fixed N_2_ under a given set of environmental conditions, the co-selection of all three main contributors, i.e., plant genotype, microbe and soil is indispensable. The inherent genetic variability of genotypes and high indigenous soil population is known to contribute significantly in nodulation and yield sometimes out-competing the inoculants strains. The chickpea plants exhibited substantial interaction between genotype/varieties, bacterial strains and soil type. Genotype and soil were found to be the most important factors contributing toward the plant response to bacterial inoculation.

## Conclusions

Being a highly nutritive cheap food, chickpea occupies a special position among legumes but mostly grown on the marginal lands by resource-poor farmers. The field analysis of PGPR inoculation to some of the elite kabuli and desi cultivars developed in the country shows that both genotypes differentially respond to inoculation under nutrient-poor and nutrient-rich soils. The data suggests co-selecting the chickpea genotypes/cultivars along-with the highly efficient rhizobial and PGPR strains for improvement of BNF efficiency and yield in chickpea. This study has demonstrated the plant beneficial potential of *O. ciceri* strain Ca-34^T^ as single or multi-strain inoculum both in marginal and fertile soil. This quality makes it an excellent candidate for development of inoculum in combination with an efficient chickpea nodulating strain to get sustainable production of both genotypes of chickpea under nutrient-poor/rich soils.

### Conflict of interest statement

The authors declare that the research was conducted in the absence of any commercial or financial relationships that could be construed as a potential conflict of interest.

## Footnotes

^1^FAO, 2014. www.fao.org.
