# Supplementary material for: Differential response of kabuli and desi chickpea genotypes toward inoculation with PGPR in different soils
Source: Front Microbiol. 2015 Aug 25;6:859. doi: 10.3389/fmicb.2015.00859 (PMC4548240; doi:10.3389/fmicb.2015.00859)
Supplement: Supplementary file 1 [file Table1.DOCX]

**Supplementary Table 1: Tukey HSD all-pairwise comparisons test for treatments (averaged over genotypes and replicates) on chickpea early growth (60DAS) and final harvest (180 DAS) compared in two soils**

|  | **Harvested 60 days after sowing** | | | | | | **Harvested 180 days after sowing** | | | | | | |
| --- | --- | --- | --- | --- | --- | --- | --- | --- | --- | --- | --- | --- | --- |
| Treatments | Shoot length  (cm) | Root length  (cm) | Shoot fresh wt.(g) | Root fresh wt. (g) | Shoot dry wt (g) | Root dry wt (g) | Plant height at harvest (cm) | No. of primary  branches | No. of secondary branches | Pods per plant | Seeds  per plant | Straw  wt. per  plant (g) | Grain wt. per plant (g) |
| **SITE 1** | | | | | | | | | | | | | |
| ***T1*** | 19.93^b^ | 15.27^b^ | 4.66^b^ | 2.04^a^ | 0.93^b^ | 0.86^a^ | 47.47^ab^ | 6.22^a^ | 8.87^a^ | 47.52^ab^ | 63.80^ab^ | 11.33^b^ | 12.75^ab^ |
| ***T2*** | 22.48^a^ | 15.18^b^ | 6.03^a^ | 2.04^a^ | 1.21^a^ | 0.87^a^ | 48.42^ab^ | 6.12^a^ | 9.45^a^ | 45.37^b^ | 63.37^ab^ | 11.47^b^ | 12.65^ab^ |
| ***T3*** | 22.82^a^ | 15.22^b^ | 5.93^a^ | 2.03^a^ | 1.19^a^ | 0.87^a^ | 49.07^a^ | 6.45^a^ | 10.35^a^ | 55.10^a^ | 76.27^a^ | 14.05^a^ | 15.44^a^ |
| ***T4*** | 22.47^a^ | 19.73^a^ | 5.86^a^ | 2.13^a^ | 1.17^a^ | 0.93^a^ | 45.37^b^ | 5.67^a^ | 8.75^a^ | 42.67^b^ | 55.05^b^ | 10.14^b^ | 11.51^b^ |
| ***L.S.D (P<0.05)*** | 1.354 | 1.4777 | 0.8589 | 0.3730 | 0.1712 | 0.1616 | 3.067 | 1.223 | 2.037 | 8.705 | 12.928 | 1.898 | 2.760 |
| ***S.E*** | 0.5273 | 0.5753 | 0.3444 | 0.1452 | 0.0667 | 0.0629 | 1.1718 | 0.467 | 0.778 | 3.325 | 4.938 | 0.725 | 1.054 |
| **SITE 2** | | | | | | | | | | | | | |
| ***T1*** | 23.15^a^ | 20.67^a^ | 7.17^ab^ | 4.52^a^ | 1.47^ab^ | 1.94^a^ | 51.62^a^ | 6.35^a^ | 9.15^a^ | 57.12^a^ | 75.82^a^ | 11.60^a^ | 17.59^a^ |
| ***T2*** | 22.65^a^ | 20.62^a^ | 6.51^b^ | 3.66^b^ | 1.30^b^ | 1.57^b^ | 53.62^a^ | 6.85^a^ | 10.17^a^ | 59.42^a^ | 79.17^a^ | 14.02^a^ | 19.10^a^ |
| ***T3*** | 23.22^a^ | 20.13^a^ | 7.49^a^ | 4.56^a^ | 1.50^a^ | 1.98^a^ | 52.62^a^ | 7.05^a^ | 9.07^a^ | 58.80^a^ | 76.55^a^ | 13.23^a^ | 17.93^a^ |
| ***T4*** | 23.62^a^ | 18.93^a^ | 7.63^a^ | 4.12^ab^ | 1.52^a^ | 1.74^ab^ | 51.30^a^ | 6.90^a^ | 9.12^a^ | 56.15^a^ | 73.05^a^ | 12.99^a^ | 15.60^a^ |
| ***L.S.D (P<0.05)*** | 1.1611 | 1.7596 | 0.8918 | 0.6592 | 0.1823 | 0.2829 | 3.061 | 1.342 | 2.394 | 12.696 | 18.105 | 2.839 | 4.090 |
| ***S.E*** | 0.4521 | 0.6851 | 0.3472 | 0.2567 | 0.0710 | 0.1101 | 1.169 | 0.512 | 0.914 | 4.849 | 6.915 | 1.084 | 1.562 |

*The means followed by the same letters do not differ significantly at α=0.05 (LSD), S.E= Standard error*

**Supplementary Table 2: ANOVA table of the effect of bacterial inoculation on early growth (60DAS) and final harvest (180 DAS) of chickpea compared in two soils *(Showing the probability values at 0.05 level)***

|  | **Harvested 60 days after sowing** | | | | | | **Harvested 180 days after sowing** | | | | | | |
| --- | --- | --- | --- | --- | --- | --- | --- | --- | --- | --- | --- | --- | --- |
| Treatments | Shoot length  (cm) | Root length  (cm) | Shoot fresh wt.(g) | Root fresh wt. (g) | Shoot dry wt (g) | Root dry wt (g) | Plant height at harvest (cm) | No. of primary  branches | No. of secondary branches | Pods per plant | Seeds  per plant | Straw  wt. per  plant (g) | Grain wt. per plant (g) |
| **SITE 1** | | | | | | | | | | | | | |
| **Genotypes (G)** | 0.0000* | 0.0433* | 0.0003* | 0.0110* | 0.0003* | 0.0088* | 0.0000* | 0.0146* | 0.0243* | 0.0000* | 0.0000* | 0.0000* | 0.0000* |
| **Treatments (T)** | 0.0000* | 0.0000* | 0.0001* | 0.8568 | 0.0001* | 0.7187 | 0.0000* | 0.0014* | 0.0004* | 0.0025* | 0.0006* | 0.0000* | 0.0030* |
| **G x T** | 0.6215 | 0.9250 | 0.9487 | 0.6408 | 0.9386 | 08086 | 0.2955 | 0.6589 | 0.6653 | 0.0000* | 0.0000* | 0.0000* | 0.0000* |
| **Minimum** | 17.667 | 14.875 | 4.300 | 1.5812 | 0.8613 | 0.6800 | 11.708 | 0.3183 | 0.0767 | 19.312 | 19.62 | 6.196 | 5.169 |
| **Maximum** | 28.542 | 17.958 | 7.678 | 2.7187 | 1.5333 | 1.1679 | 32.667 | 0.5842 | 0.1408 | 81.938 | 114.00 | 18.689 | 21.472 |
| **SITE 2** | | | | | | | | | | | | | |
| **Genotypes (G)** | 0.0000* | 0.0440* | 0.0000* | 0.0014* | 0.0000* | 0.0030* | 0.0000* | 0.0000* | 0.0003* | 0.0000* | 0.0004* | 0.0039* | 0.0000* |
| **Treatments (T)** | 0.2067 | 0.0438* | 0.0071* | 0.0015* | 0.0083* | 0.0008* | 0.0010* | 0.0302* | 0.0432* | 0.9012 | 0.8503 | 0.1664 | 0.9012 |
| **G x T** | 0.0325* | 0.0043* | 0.2110 | 0.0000* | 0.2286 | 0.0000* | 0.3353 | 0.0973 | 0.0813 | 0.0027* | 0.0010* | 0.0312* | 0.0027* |
| **Minimum** | 19.833 | 18.500 | 5.424 | 3.2142 | 1.0883 | 1.3817 | 23.542 | 0.4071 | 0.0975 | 41.567 | 44.375 | 9.351 | 12.318 |
| **Maximum** | 30.583 | 41.625 | 10.545 | 5.4475 | 2.1054 | 2.3458 | 43.208 | 0.6800 | 0.1617 | 85.875 | 97.050 | 16.343 | 22.307 |

** Highly significant at 0.05%*
